# Supplementary figures and images for: Path for recovery: an ecological overview of the Jambato Harlequin Toad (Bufonidae: Atelopus ignescens) in its last known locality, Angamarca Valley, Ecuador
Source: PeerJ. 2024 Jun 21;12:e17344. doi: 10.7717/peerj.17344 (PMC11195548; doi:10.7717/peerj.17344)

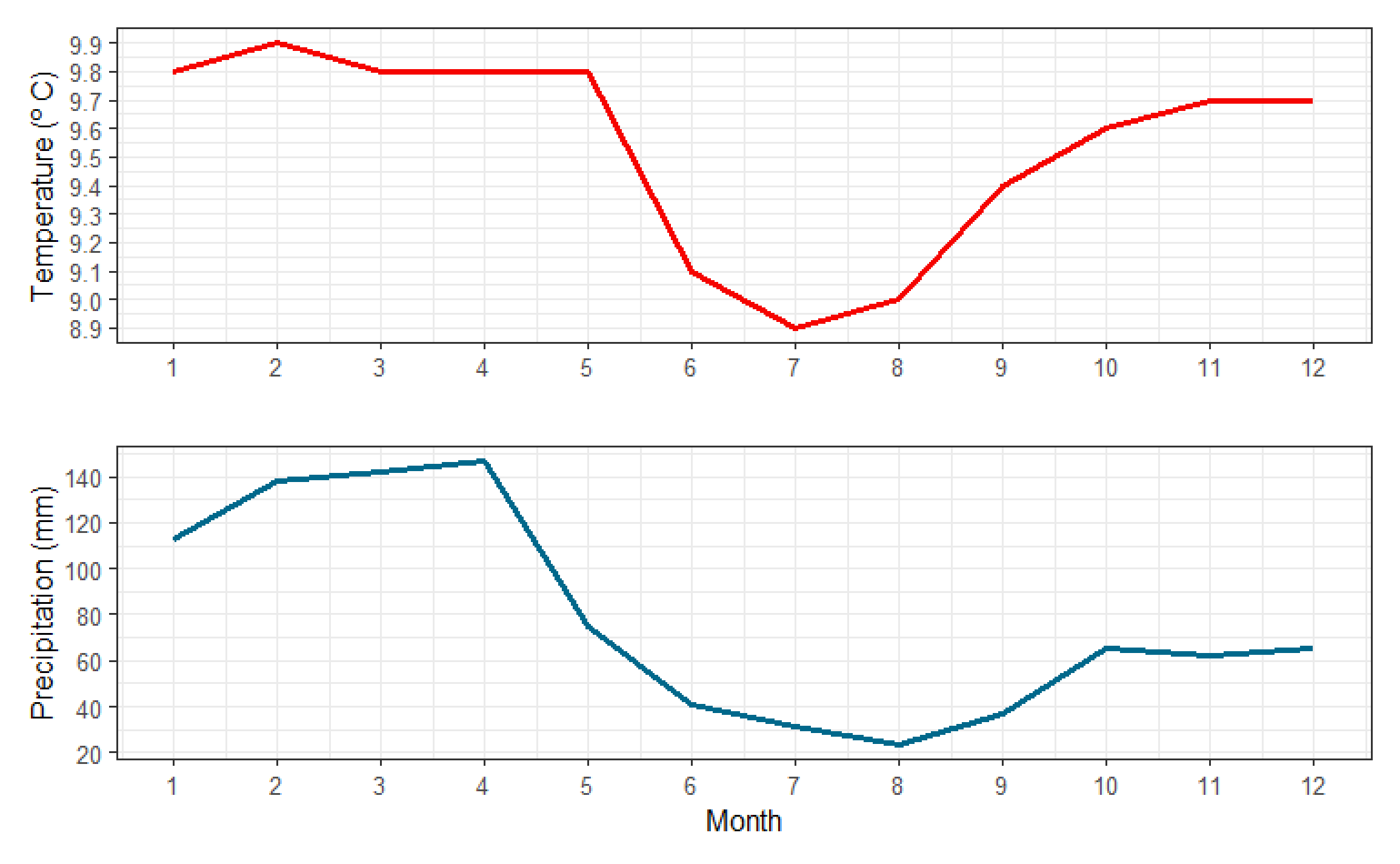

Supplement: Supplemental Information 1 — Image prepared by Mateo A. Vega-Yánez. [file peerj-12-17344-s001.png]
